# Implementing research results in clinical practice- the experiences of healthcare professionals

**DOI:** 10.1186/s12913-016-1292-y

**Published:** 2016-02-10

**Authors:** Nanna Kristensen, Camilla Nymann, Hanne Konradsen

**Affiliations:** Gentofte Hospital, Kildegårdsvej 28, 2900 Hellerup, Denmark

**Keywords:** Implementation science, Implementation of research results, Implementation in hospital settings, Implementation process

## Abstract

**Background:**

In healthcare research, results diffuse only slowly into clinical practice, and there is a need to bridge the gap between research and practice. This study elucidates how healthcare professionals in a hospital setting experience working with the implementation of research results.

**Method:**

A descriptive design was chosen. During 2014, 12 interviews were carried out with healthcare professionals representing different roles in the implementation process, based on semi-structured interview guidelines. The analysis was guided by a directed content analysis approach.

**Results:**

The initial implementation was non-formalized. In the decision-making and management process, the pattern among nurses and doctors, respectively, was found to be different. While nurses’ decisions tended to be problem-oriented and managed on a person-driven basis, doctors’ decisions were consensus-oriented and managed by autonomy. All, however, experienced a knowledge-based execution of the research results, as the implementation process ended.

**Conclusion:**

The results illuminate the challenges involved in closing the evidence-practice gap, and may add to the growing body of knowledge on which basis actions can be taken to ensure the best care and treatment available actually reaches the patient.

**Electronic supplementary material:**

The online version of this article (doi:10.1186/s12913-016-1292-y) contains supplementary material, which is available to authorized users.

## Background

Healthcare research continually produces large amounts of results and revised methods of treatment and care for patients, which, if implemented in practice, can potentially save lives and improve the quality of life of patients [1]. Nonetheless, a rise in the amount of research results available does not automatically translate into improved patient care and treatment [2, 3].

There is broad evidence that there is a substantial gap between the healthcare that patients receive and the practice that is recommended – also known as the research-practice gap, evidence-practice-gap or knowing-doing gap [4–6]. Evidence suggests that it sometimes takes more than a decade to implement research results in clinical practice, and that it is often difficult to sustain innovations over time [7, 8]. This is critical, not only for patients, who thereby fail to receive the best treatment and care available, but also for healthcare organizations and society, who miss out on the potential financial value gains and returns on investment [9, 10].

Implementation Science is the mail field of research dedicated to exploring methods of implementing research evidence into practice [11, 12]. Many studies within this field explore methods to promote integration of research findings by policymaking and through larger, systematic and planned implementation initiatives such as e.g. Consolidated Framework For Implementation Research (CFIR) [13]. Fewer studies unfold whether and how research results seems to wander into practice in a less structured, planned and top down manner through local, emerging and personally carried mechanisms.

Within the field of translational science (the translation of new clinical knowledge into improved health) studies suggesting methods for bridging the gap between research and practice [14] mainly focus on exploring implementation methods capable of promoting the exchange, transfer, diffusion and dissemination of evidence-based knowledge to practitioners and decision-makers in healthcare systems [14]. Journal clubs are similarly a widespread dissemination method for clinicians to access evidence-based knowledge through presentations and discussions of research articles [15]. What these approaches have in common is a focus on how to convey evidence-based information to healthcare professionals, and thereby raise awareness of relevant improvements in treatment and care. A large body of research nonetheless suggests that it is difficult for professionals to utilize new, decontextualized, explicit knowledge in their daily work practice [16–18]. What directs the professional’s actions in practice will often be the implicit and established know-how of routines– even when decisions on new methods and the commitment to put them into practice is otherwise present [19–21].

In line with these insights, translational methods have been shown to produce only small to moderate effects, and research suggests that the successful uptake of research results in the actions of healthcare professionals requires more than merely making the results accessible for local practice [3, 22, 23].

Recent research suggests that evidence-informed practice is mediated by an interplay between the individuals, the new knowledge and the actual context in which the evidence is to be operationalized and utilized in daily practice [24, 25]. Organizational contextual factors such as culture and leadership, but also social and attitudinal factors as professional opinion has shown to have a great impact on implementation success [12, 26, 27]. In this perspective, new research results are not transferred on a 1:1 basis from academia to practice. Instead, the applicability of research results must be locally evaluated, and new results must eventually be made actionable and utilizable, and adapted to local practice, in order to produce the desired outcome over time [22, 23, 27-29].

Deepening our understanding of the factors which prohibit or promote this interplay in local practice and the operationalization and use of research results in daily clinical life is vital in order to bridge the continuing gap between healthcare research and practice [30, 31].

The objective of this study is to elucidate how healthcare professionals in a hospital setting experienced working with the implementation of research results in practice, and which existing methods they utilized to incorporate research results into daily healthcare action.

## Method

### Design

A descriptive qualitative design was chosen, as the aim of the study was to elucidate the experiences of healthcare professionals. A directed content analysis approach guided the analysis [32].

### Setting and participants

The participants were healthcare professionals working in two different medical wards in a medium-sized university hospital in Denmark. In order to capture viewpoints representing various different roles in the implementation process, the following professionals from each ward were invited to participate (Table 1).

**Table 1:** (Participant characteristics)

Participant	Ward 1	Ward 2
Medical Head of Department	x ^a^	x
Senior Physician	X	x ^a^
Nursing Head of Unit	X	x
Doctor	X	X
Nurse	X	X
Physician with special research responsibility	x ^a^	x ^a^
Nurse with special research responsibility	X	X

^a^Dual role filled by the same person

As there was an overlap between the positions in two instances, twelve interviews were carried out. The wards were selected on the basis of having several researchers employed, as well as their willingness to participate.

The participants were recruited through the heads of departments, who were asked to identify professionals eligible to participate. A calendar invitation was subsequently sent out inviting the professionals to participate, and all agreed.

### Data collection

Data was collected in the spring of 2014 through 12 qualitative, semi-structured interviews. All of the interviews took place in the wards. The theoretical framework of Klein & Knight [33] served as the basis of the interview guide (see Additional file 1).

The theoretical framework consisted of factors enhancing implementation: 1) a package of implementation policies and practices established by an organization, 2) the climate for innovation implementation in the team or organization —i.e. the employees’ shared perceptions of the importance of innovation implementation within the team or organization, 3) managerial support for innovation implementation, 4) the availability of financial resources, 5) a learning orientation: a set of interrelated practices and beliefs that support and enable employee and organizational skill development, learning, and growth, 6) managerial patience, i.e. long-term orientation. The framework also consisted of the following challenges to implementation: 1) many technological innovations are unreliable and imperfectly designed, 2) many innovations require would-be users to acquire new technical knowledge and skills, 3) the decision to adopt and implement an innovation is typically made by those higher up in the hierarchy than the innovation’s targeted users, 4) many team-based and organizational innovations require individuals to change their roles, routines, and norms, 5) implementation is time-consuming, expensive, and, at least initially, a drag on performance, and 6) organizational norms and routines foster maintenance of the status quo.

The opening question of the interviews was always open-ended, asking the participants to talk about their own experiences of working with research implementation in practice. Consequently, the participants contributed as much detailed information as they wished, and the researchers asked further questions as necessary. The interviews lasted on average 45 min, and were conducted by the first and second author of this article. One person acted as the main interviewer while the other observed the interview as a whole, ensuring follow-up in accordance with the interview guide. All interviews were recorded and transcribed.

### Data coding and analysis

A directed and deductive content analysis approach [34] guided the analysis in order to bring theoretically-derived coding categories into connection with the empirical data.

Transcripts were entered into NVivo10 in order to structure the data. An unconstrained categorization matrix was developed on the basis of the twelve theoretical factors to guide the analysis, as described by Elo et al. [34]. Data was coded according to the categories in the matrix. During the coding process, new categories emerged, such as issues about professionals using their spare time for research and research implementation, and multidisciplinarity among doctors and nurses. The new categories were noted and treated as equally important additions to the initial categories. Once the coding process was complete, the number of categories was reduced by “*collapsing those that are similar or dissimilar into broader higher-order categories.*” [34], while maintaining proximity to the text. This abstraction process was repeated with the higher-order categories, resulting in six main categories, as described in the results section of this article.

In order to enhance rigor and validity, interviews were initially coded by all authors individually, after which they met and discussed the categorization until consensus was obtained [35].

The manuscript adheres to the RATS Guidelines in order to enhance credibility.

### Ethical considerations

The study was submitted to *The Committees on Health Research Ethics for the Capital Region of Denmark (De Videnskabsetiske komiteer for Region Hovedstaden),* who assessed that the study did not require formal approval by the committee.

As only two wards at the hospital served as the empirical basis of the study, the researchers paid special attention to issues of confidentiality and anonymity. Participants were therefore informed that their names would not be mentioned in the study, but were also asked to reflect on the fact that the limited number of participants might make it difficult to maintain total anonymity. With this information in mind, all participants gave their written, informed consent prior to participating.

## Results

In this study of the experience of healthcare professionals with existing ways of incorporating research results into healthcare action, six main categories were identified: *non-formalized*, *consensus-oriented*, *problem-oriented*, *autonomous, person-driven* and *knowledge-based.*

**Fig. 1:**
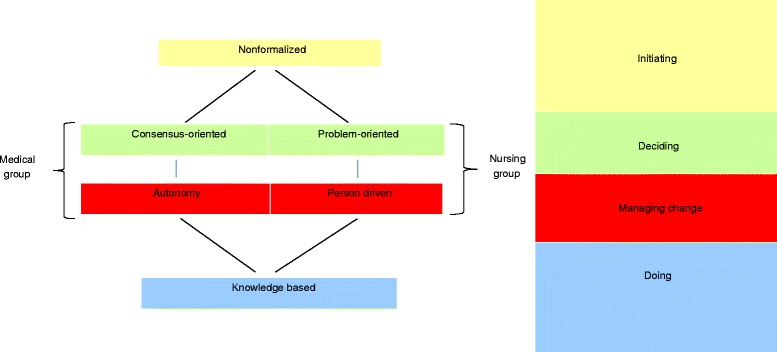
Activities and stages in the process of implementation of research results in clinical practice

These main categories related in different ways to the varying implementation activities of *initiating, deciding on, managing* and *executing* change. These activities are associated with different stages in the process of implementation, and the main categories are therefore structured around these (Fig. 1).

The healthcare professionals experienced no formalized procedures or established workflows in relation to initiating the implementation of research results. One nurse explained how the work of initiating implementation was not integrated into the conclusion of a research project:

*“When we have the results, we are prone to think ‘Well, that’s that’ and move on to a new project.” (Nurse)*

In relation to describing, searching for, remaining updated on, and evaluating the relevance of new research knowledge within various areas, one doctor commented:

*“…there is no system in it. It’s not as though we say, ‘you keep an eye on what’s going on in this field, and I’ll keep an eye on this’. It becomes somewhat unsystematic.” (Doctor)*

No well-defined assignments, roles or responsibilities emerged in the experience of translating research results into practical implementation. One doctor described the uncertainty experienced due to the lack of a more systematic approach to research implementation, and stated that:

*“…I think it would be nice to have some kind of system or safety net, so that you don’t have to worry about whether something may have slipped through.” (Doctor)*

Heads of departments and heads of units were not active in initiating the implementation of new research in practice. Their participation was mostly limited to approving, allocating resources or applying for financial support when a healthcare professional wished to initiate the implementation of a research result.

Often, highly-motivated persons with specific research interests took the initiative into their own hands to suggest the implementation of certain new research results in practice. In the nurse group this was often a clinical nurse specialist, whereas in the doctor group any doctor might initiate a potential implementation. One senior physician with special research responsibility described this as being closely related to a high degree of motivation to take action:

*“…people take things seriously. They don’t just sit around and wait for an instruction to arrive. I mean – they just do things.” (Physician with special research responsibility)*

This informal practice of individuals independently initiating the implementation of research results was also seen in doctors putting in extra hours after their formal working hours, both to conduct their own research and to acquire the skills necessary to implement a certain new result in practice, such as a surgical technique or a new item of equipment. In this connection, both research and the implementation of research were to some extent driven by individual interests and motivation that went beyond formal obligations.

When deciding on and managing implementation, various patterns were described in relation to doctors and nurses. In the doctor group, the decision to implement a new result was described as a consensus process among the senior physicians; managing implementation, on the other hand, was experienced as being regulated by individual doctors autonomously.

New research results were discussed at weekly meetings between all doctors – either on the basis of the doctors’ own research, articles, or input from external conferences. The most specialized physicians within a clinical area selected and presented new research results to their colleagues. These presentations were followed by discussion in the group – sometimes debating the results, and at other times considering whether to implement the results in practice or not. One doctor said:

*“If there is some kind of consensus that this sounds exciting and this is something that we would like to proceed with, then we can agree that it is something we will do.” (Senior physician)*

In this way a consensus decision was arrived at, and the group would then define the principles for implementing new methods of patient treatment. As one doctor described it:

*“We discuss it in the group and agree that to begin with, we will apply a strategy in which we only do this with some (patients).” (Physician with special research responsibility)*

Once a consensus decision had been taken to implement a new result, the collective coordination ceased, and was replaced by a principle of the individual autonomy of each doctor to manage his or her own decisions in practice.

“*We have a high degree of autonomy. We do not all do things the same way.*” (Senior Physician)

In this way, any doctor could refrain from implementing a new practice that had been decided on in the group, or act on something that had not been decided in the group, without the need to ask permission. Due to autonomy, no organized follow-up was conducted from within the ward to manage and monitor the implementation of research results. Some healthcare professionals said that if there was a follow-up on the implementation of a new research result, it was often in practice conducted by agents from the pharmaceutical industry who wished to establish the application of certain products.

The principle of autonomy was also visible in deciding whether to adopt new instructions and guidelines. As a head of ward stated:

*“…we don’t have to follow these guidelines. There may be many other issues to consider. We might be satisfied with the treatment that we already have, and not find the new treatment much better. It might even be more expensive. So we don’t have to put it into practice.” (Medical Head of Department)*

In the doctor group, therefore, the experience of deciding on and managing the implementation of research results in practice showed both an orientation towards achieving consensus decisions, and at the same time a principle of managing change autonomously in practice.

Fewer persons participated in decision-making and management in the nurse group. One or more clinical nurse specialists and a Nursing Head of Unit jointly planned a process to collect research results and design an intervention to change practice. Most of the time, the proposals came from clinical specialists, with the formal aim of remaining updated on research within the overall professional field. One clinical specialist said:

*“…our practice was very individual and experimental, so I made a search for the existing evidence. And on that basis I implemented a new practice.” (Nurse with special research responsibility)*

A problem in existing practice inspired clinical nurse specialists to revise that practice, and on that basis seek out existing research results. Clinical nurse specialists were seen as agents of change with responsibility to manage the implementation of a research result as a revised practice in cooperation with the Nursing Head of Unit. Clinical nurse specialists were referred to as ‘key persons’ and ‘resource persons’ who searched for evidence, designed the changes in practice, coordinated changes with other sections, produced instructions and maintained focus on the change at status meetings. The clinical nurse specialists also performed a supervisory role in relation to updating nurses’ knowledge, training their skills in practice, monitoring whether nurses carried out the new practice, and addressing those nurses who failed to adopt the new methods or experienced problems with them. One nurse with special research responsibility described how she worked to spread change in the nursing group by engaging particularly motivated staff members to advocate the new practice and act as change ambassadors in the daily routine:

*“You have to have someone, either yourself, or someone else that you identify, to continually stir the pot. Saying ‘we are all responsible’ is the same as saying ‘no-one takes responsibility’.” (Nurse with special research responsibility)*

At the same time, both clinical nurses and Nursing Heads of Unit described instances when the implementation of research results failed because nobody took action on the agreed plan. Despite the intention to implement the change, the clinical specialists described how they failed to actually turn decisions about changes into revised practice. One nurse explained:

*“We can easily agree – but the motivation falls away as we walk out of the room, because other assignments accumulate for the resource persons that have been in the room, such as the need to cover a shift.” (Nurse)*

Failure to allow time to follow up and anchor new decisions in practice was experienced as the result of competing agendas overruling the management of the decisions, as ‘there is simply no room for more’. Both the large flow of patients, the pressure to keep up efficiency figures and the large number of other, unrelated implementation processes going on in connection with quality improvement were seen as barriers to implementing the research-based changes.

*“…the combination of the high level of pushing patients through the hospital – that is, the high demands towards production – and the great focus on efficiency conflict with the need to conduct that many development projects, each with their own requirements, and at the same time.” (Nurse)*

The overriding focus on production was experienced as being closely related to management focus and behavior:

*“Management – the senior management here – is very focused on the bottom line figures. There is definitely a lack of follow-up and a lack of people insisting ‘now we’re going to do this’.” (Nurse with special research responsibility)*

As well as being the ones with the responsibility for managing changes, nurses with special research responsibility also saw themselves as being very much alone and having trouble making the changes on their own:

*“I’m the only one who is trained to read research articles. And that’s not enough – there is not enough discussion to be able to drive a change. There is not enough resonance. I’m on my own.” (Nurse with special research responsibility)*

Operationalizing research results into revised action in practice was mainly knowledge-based, in terms of generating information external to the individuals handling the knowledge [36]. Moving from the decision to executing the changes was mostly experienced as a procedure to create a new instruction or a supplement to an existing one.

*“The procedures to be changed are written down in instructions on how to perform them.” (Medical Head of Department)*

When reflecting on how this was done, one doctor stated:

*“…a document, an instruction is created, and from there on everybody does it.” (Doctor)*

In this respect there was a widespread belief that changes would emerge from written words/documents. Information-sharing about decisions took place between a few consultant doctors in the doctor group. As one doctor said:

*“Actually, only a very small group needs to know where we are going. Because then you pass it on to others.” (Doctor)*

The actual implementation took place randomly, as senior physicians ‘passed it on’ to junior doctors when approving professional decisions in relation to the treatment of patients. When reflecting on how the new information reaches the majority of other healthcare professionals, one Medical Head of Department stated:

*“Well, you’re obliged to read the instructions when you get hired in this department. They are accessible on our local network. Or on the hospital network. You can read all of them there.” (Medical Head of Department)*

At the same time as relying on knowledge-based implementation through mandatory reading of written documents, the large number of written procedures was also experienced as something that hindered healthcare professionals from knowing how to carry out the practice. Several instructions and guidelines referred to the same practice, and reading all of the instructions simultaneously was experienced as too demanding in a busy schedule, resulting in a failure to read them.

Other types of knowledge-based implementation included exchanging and sharing information at meetings, and in newsletters and e-mails. Both doctors and nurses described teaching each other theoretically, sharing knowledge, and in some cases attending formal training, such as conferences or courses.

Nonetheless, these practices were seen as ineffective in implementing research results. As one nurse expressed it:

*“Of course you can inform people, teach them – but it changes nothing.” (Nurse)*

Applying job training and bedside learning in the implementation of research results was common in the nurse group. As one clinical nurse specialist explained her practice:

*“I had a list of the people who had received the theoretical teaching, and what we did was that they accompanied me with a patient and observed me showing them ‘how to do it’, and then I observed them, so that I could supervise them.” (Nurse with special research responsibility)*

On-the-job training was perceived as being a more efficient way of implementing research results, but at the same time much more demanding on resources:

*“My experience is that face-to-face is the way to do it, to create the understanding, as well as ensuring their ability to do it afterwards. But it’s time-consuming.” (Nursing Head of Unit)*

Doctors also referred to on-the-job training in relation to the implementation of new procedures. In such cases one or more of the doctors acquired a new skill and then taught it to colleagues who were interested, but no organized on-the-job training was described.

## Discussion

The objective of this study has been to elucidate how healthcare professionals in a hospital setting experienced working with research implementation in practice, and which existing methods of incorporating new research results into daily healthcare action they had experienced. This is not the first study to explore experiences of healthcare professionals with implementation of research results, however most other studies, examine how researchers and policymakers might work with clinicians rather that how clinicians work on their own. This study suggests that clinicians *do* work intentionally implementing research results by themselves. And knowing the mechanisms regulating this intentional implementation effort is important in furthering the knowledge on how to ensure a best practice patient care and evidence based healthcare systems.

We found the initiation of the implementation of research results to be largely non-formalized at the organizational level and not led by management. According to the literature, refraining from formalizing which research results are to be implemented, and how they are to be implemented, can both benefit and compromise the operationalization of research results in healthcare practice.

In a longitudinal case study of healthcare improvement, Booth et al. [37] argued that improvements in chronic illness care emerged as a result of co-evolution, non-linearity and self-organization in a network of agents, and not as a result of planned system change. In this view, the non-formal character of research implementation could be thought to provide a necessary space of possibilities for improvements to emerge. The non-formalized nature of implementation also provides plenty of room for individual initiative and opportunities to define which research results are to be implemented and how they are to be implemented. This participation and self-determination is argued to be vital to securing an affective commitment to the changes and an intrinsic motivation to change clinical behavior [38, 39].

On the other hand, formal leadership is claimed to be an important ingredient in making changes happen in practice, particularly in terms of obtaining the same affective commitment to change on the part of organization members [40]. Stelter et al. [41] argue that supportive leadership behaviors – including articulating visions, clarifying prioritized goals, handling inhibitors in the implementation process, etc. – are necessary to institutionalize evidence-based practices in an organization. According to Birken et al. [42], middle managers are crucial in the area of diffusing and synthesizing information, selling the innovation and mediating between strategy and day-to-day activities. Furthermore, the lack of formal procedures and overall professional-strategic steering may risk failure to prioritize the limited resources in areas where improvements in healthcare and patient treatment are most needed [43, 44], systematic and prioritized knowledge implementation efforts need not exclude the possibility of aspiring, well-motivated and self-determined healthcare professionals creating bottom-up changes. By promoting ‘enabling contexts’, managers can support emerging processes with visionary proposals and commitment, while professionals can generate innovation and professional development within strategically selected areas [44].

In relation to deciding on and managing change, nurses and doctors in the study followed different patterns.

The consensus approach, as a decision-making method, has also been studied by others [45]. Investigating the process of practice change in a clinical team, Mehta et al. (1998) found that in addition to evidence-based knowledge, group dynamics, the process of dialogue and personal experience also exerted a considerable influence on the consensus that was reached and the decisions that were made [44]. In this perspective, multiple, non-formal and non-rational factors can influence the decisions that are taken in clinical teams in relation to initiating and deciding to implement research results.

In the management of the changes in practice, the autonomy of individual doctors was key. Our results indicated that even though decisions were reached through consensus, they were not perceived as binding in clinical practice. Clinical autonomy is a phenomenon that is well described elsewhere in the literature [46]. A recent study showed that doctors preferred their individual, non-systematic professional assessments to a formalized strategic approach in which they applied the recommendations of a large, predetermined, evidence-based program when treating COPD patients across sectors [47].

Armstrong [48] has described this preference for autonomy as a defense of professional identity and the right to act independently without instructions from others. One interpretation of clinical autonomy is that the individual practitioner strives to preserve traditional privileges and monopolies of knowledge in the medical profession – e.g. by using the rhetoric of a patient-centered treatment that allows the professional to act autonomously and avoid the constraints of professional control and standardization.

Another interpretation of the phenomenon is that clinical autonomy is a necessary prerequisite for doctors to be able to make research-based decisions on the treatment of individual patients, since such decisions are not only based on clinical evidence, but also on factors such as patient preference and clinical expertise [49, 50]. In this perspective, the lack of formalization and management of professional development, as our results also indicate, can be viewed as a valuable and desirable aspect of maintaining professional healthcare practice on the basis of the individual doctor’s expertise [51].

The implementation of research results in the nurse group was driven and managed by a single nurse or a small group of nurses, with a clinical nurse specialist in each ward as key to managing the changes. One could argue that these nurses play a role that is somewhat similar to the role of nurse facilitators as described by Dogherthy et al. [52]. According to Dogherthy, the nurse facilitator is an important element in advancing evidence-based nursing practice. The role is very broadly defined in the literature as ranging from specific task-driven actions to releasing the inherent potential of individuals [52]. However, one common denominator that is stressed in order for the nurse facilitator to succeed in implementing research results is the deputizing of the nurse facilitator. Lack of authority has in several studies been identified as a barrier to the implementation of research results by nurses [52–54].

The background and competencies of nurses leading change processes on the basis of research results is argued to be important in determining whether their management of research implementation will succeed. Currey et al. [55] argue that nurses who facilitate evidence-based clinical practice must have a doctorate, be recognized clinical experts with educational expertise and advanced interpersonal, teamworking and communications skills. In line with this research-active nurses have been found to be more likely to overcome barriers in the implementation process and to succeed in translating research into practice [56].

Having only a few research-active nurses in a ward thus seems to be a barrier for implementation in itself, as the majority of nurses in the wards abstained from using evidence-based knowledge, giving three typical reasons: lack of time, lack of interest and lack of qualifications [56].

All in all, considering the competencies and the available management space of key-persons/clinical nurse specialists, leadership of the implementation of research results seems to be key to the implementation of research results in clinical practice.

The manner of executing the implementation of new research results in practice resonates with the tradition in translational science of focusing on conveying evidence-based information in the implementation of research results in practice [14].

We found widespread use of written clinical instructions, guidelines and newsletters when executing the implementation of research, as has also been found in other studies on implementation methods in healthcare [22].

It has been argued in several studies that issuing clinical guidelines, etc., serves to protect the collective professional autonomy from administrative pressures by clearly demonstrating a commitment to high standards of care, thereby justifying professional independence [57, 58].

When considering the knowledge-based approach, studies found that the mere dissemination of evidence-based clinical guidelines was ineffective in changing the behavior of healthcare professionals [2, 59, 60]. In a decision science study, Mohan et al. [61] demonstrated doctors’ non-compliance with clinical practice guidelines in trauma triage. 80 % of the doctors failed to refer trauma patients to trauma centers, even though the patients met the guideline conditions for transfer. The authors attribute the non-compliance to non-evidence-based attitudes on the part of the doctors, plus organizational norms and incentives that influenced the doctors’ perceptions and decision-making procedures.

Creating behavioral compliance with the content of guidelines has been shown to require the continuous involvement of all staff in establishing new routines to utilize the guidelines [62]. Our results indicated no such involvement of general staff, as one or a few professionals often developed or discussed the guidelines independently. The widespread belief that creating new guidelines and relying on the mandatory reading of these will in itself change the behavior of healthcare professionals may be a barrier to the effective implementation of new research results in practice.

With regard to managing implementation though e-mails, classroom teaching, etc., Rangachari et al. [3] have described these methods as ineffective in establishing new research-based practice as they solely raise awareness of the change, but fail to make it actionable.

Other researchers also point to the importance of making knowledge actionable [63]. The transformation of explicit knowledge and awareness into new skills may very well depend on activities such as acting out and improvising, mentally and motorically, the new intentions and knowledge, and thereby operationalizing and internalizing the knowledge and continually regulating actions to produce the desired outcome [16, 64].

Opportunities to act upon new research results in practice were scarce in our results, and more common among the nurses than among the doctors. However, in both cases there were reports of on-the-job training and bedside learning, which were perceived as being more effective in changing the behavior of staff in the ward.

### Limitations

The use of interviews to investigate the implementation processes may have influenced the data and the linear change model that emerged from the interviews. When invited to articulate their experiences of research implementation, participants may have generated narratives with a beginning, a middle and an end, whereas more complex and circular processes may have taken place.

Investigating implementation practice through additional methods such as observational studies, participation in meetings and daily clinical practice may provide further insight into the nature of common implementation processes in healthcare systems.

## Conclusions

The experience of research implementation illuminated a process that was unsystematically initiated, relied on few stakeholders, and often ended up on paper rather than in practice and in the actual treatment and care of individual patients. The results reveal that this on the one hand describes the challenges involved in closing the evidence-practice gap, but on the other hand supports the commitment of professionals with special research interests. The results of this study will add to the growing body of knowledge on which basis action can be taken to ensure that the best care and treatment available actually reaches the patient.

## Additional file

Additional file 1:
**Interview guide (translated from Danish).** (PDF 70 kb)

## References

[1] Cummings GG, Olivo SA, Biondo PD, Stiles CR, Yurtseven O, Fainsinger RL, et al. Effectiveness of Knowledge Translation Interventions to Improve Cancer Pain Management. J Pain Symptom Manage. 2011;41:915. 10.1016/j.jpainsymman.2010.07.017 21398088

[2] Brown, D., McCormack, B. Developing Postoperative Pain Management: Utilising the Promoting Action on Research Implementation in Health Services (PARIHS) Framework. Worldviews Evid Based Nurs, Third Quarter. 2005;2(3):131–141. 10.1111/j.1741-6787.2005.00024.x 17040534

[3] Rangachari P, Rissing P, Rethemeyer K (2013). Awareness of evidence-based practices alone does not translate to implementation: insights from implementation research. Qual Manag Health Care.

[4] Pfeffer J, Sutton RI (2000). The Knowing-Doing Gap: how smart companies turn knowledge into action.

[5] Real K, Poole MS. Innovation Implementation: Conceptualization And Measurement In Organizational Research, in (ed.) Research in Organizational Change and Development (Research in Organizational Change and Development, Volume 15). Bingley: Emerald Group Publishing Limited; 2005.

[6] Lilienfeld SO, Ritschel LA, Lynn SJ, Brown AP, Cautin RL, Latzman RD (2013). The research-practice gap: bridging the schism between eating disorder researchers and practitioners. Int J Eat Disord..

[7] Dilling JA, Swensen SJ, Hoover MR, Dankbar GC, Donahoe-Anshus AL, Murad MH, et al. Accelerating the use of best practice: The Mayo Clinic model of diffusion. Jt Comm Journal Qual Saf. 2013;39(4):167–76. 10.1016/s1553-7250(13)39023-0 23641536

[8] Ploeg J, Markle-Reid M, Davies B, Higuchi K, Gifford W, Bajnok I, et al. Spreading and sustaining best practices for home care of older adults: a grounded theory study. Implement Sci. 2014;9:162. 10.1186/s13012-014-0162-4 PMC4225037 25377627

[9] Donaldson NE, Rutledge DN, Ashley J (2004). Outcomes of Adoption: Measuring Evidence Uptake by Individuals and Organizations. Worldviews Evid Based Nurs, Third Quarter.

[10] Brown MM, Brown CG, Lieske HB, Lieske PA (2014). Financial return on investment of opthalamic interventions: a new paradigm. Curr Opin Ophthalmol.

[11] Ferlie E, Fitzgerald L, Wood M (2000). Getting evidence into clinical practice: an organisational behaviour perspective. J Health Serv Res Policy.

[12] Grol R, Grimshaw J (2003). From best evidence to best practice: effective implementation of change in patients’ care. The lancet.

[13] Damschroder LJ, Aron DC, Keith RE, Kirsh SR, Alexander JA, Lowery JC (2009). Fostering implementation of health services research findings into practice: a consolidated framework for advancing implementationscience. Implement Sci.

[14] Grimshaw JM, Eccles MP, Lavis JN, Hill SJ, Squires JE (2012). Knowledge translation of research findings. Implement Sci..

[15] Ebbert JO, Montori VM, Schultz HJ (2001). The journal club in postgraduate medical education: a systematic review. Med Teach.

[16] Hacker W (2003). Action Regulation Theory: A practical tool for the design of modern work processes?. Eur J Work Organ Psy..

[17] Wierdsma A, Boonstra JJ (2004). Beyond Implementation: Co-creation in change and development. Dynamics of Organizational Change and Learning.

[18] O’Connor N, Kotze B (2008). Learning Organizations: a clinician’s primer. Australas Psychiatry..

[19] Leroy F, Ramanantsoa B (1997). The Cognitive and Behavioural Dimensions if Organizational Learning in a Merger: An Empirical Study. J Manage Stud..

[20] Choi JN, Price RH (2005). The effects of person-innovation fit on individual responses to innovation. J Occup Organ Psychol..

[21] Kothari A, Rudman D, Dobbins M, Rouse M, Sibbald S, Edwards N (2012). The use of tacit and explicit knowledge in public health: a qualitative study. Implement Sci.

[22] European Science Foundation, Forward look: Implementation of Medical Research in Clinical Practice. 2011. http://www.esf.org/fileadmin/Public_documents/Publications/Implem_MedReseach_ClinPractice.pdf. Accessed date 1 Aug 2014

[23] Boaz A, Baeza J, Fraser A (2011). Effective Implementation of research into practice: an overview of systematic reviews of the health literature. BMC Res Notes.

[24] Glasgow RE, Green LW, Taylor MV, Stange KC (2012). An Evidence Integration Triangle for aligning Science with Policy and Practice. Am J Prev Med.

[25] Rycroft-Malone J, Seers K, Chandler J, Hawkes CA, Drichton N, Allen C, et al. The role of evidence, context, and facilitation in an implementation trial: implications for the development of the PARHIS framework. Implement Sci. 2013;8:28. 10.1186/1748-5908-8-28 PMC3636004 23497438

[26] Kitson A, Harvey G, McCormack B (1998). Enabling the implementation of evidence based practice: a conceptual framework. Qual Health Care.

[27] Clancy CM, Cronin K (2005). Evidence-Based Decisionmaking: Global Evidence, Local Decicions. Health Affair.

[28] Chambers DA, Glasgow RE, Stange KC (2013). The dynamic sustainability framework: addressing the paradox of sustainment amid ongoing change. Implement Sci..

[29] Parker LE, de Pillis E, Altschuler A, Rubenstein LV, Meredith LS (2007). Balancing participation and expertise: a comparison of locally and centrally managed health care quality improvement within primary care practices. Qual Health Res.

[30] Cochrane LJ, Olsen CA, Murray S, Dupuis M, Tooman T, Hayes S (2007). Gaps Between Knowing and Doing: Understanding and Assessing the Barriers to Optimal Health Care. J Contin Educ Health Prof.

[31] Novins DK, Green AE, Legha RK, Aarons GA (2013). Dissemination and Implemenation of Evidence-Based Practices for Child and Adolescent Mental Health: A Systematic Review. J Am Acad Child Adolesc Psychiatry.

[32] Hsieh H-F, Shannon SE (2005). Three Approaches to Qualitative Content Analysis. Qual Health Res..

[33] Klein KJ, Knight AP (2005). Innovation Implementation: Overcoming the Challenge. Curr Dir Psychol Sci..

[34] Elo S, Kyngäs H (2008). The qualitative content analysis process. J Adv Nurs.

[35] Slevin E, Sines D. Enhancing the truthfulness, consistency and transferability of a qualitative study: utilising a manifold of approaches. Nurse Res. 1999/2000;7(2):79–99.

[36] Schneider U (2007). Coping with the Concept of Knowledge. Mange Learn..

[37] Booth BJ, Zwar N, Harris MF (2013). Healthcare improvement as planned system change or complex responsive processes? a longitudinal case study in general practice. BMC Fam Pract..

[38] Gagné M, Koestner R, Zuckerman M (2000). Facilitating Acceptance of Organizational Change: The Importance of Self-Determination. J Appl Soc Psychol..

[39] Nantha YS (2013). Intrinsic motivation: how can it play a pivotal role in changing clinician behavior?. J Health Organ Manag.

[40] Michaelis B, Stegmaier R, Sonntag K (2009). Affective Commitment to Change and Innovation Implementation Behavior: The Role of Charismatic Leadership and Employees’ Trust in Top Management. J Change Manag.

[41] Stelter C, Ritchie J, Rycroft-Malone J, Charns M (2014). Leadership for Evidence-Based Practice: Strategic and Functional Behaviors for Institutionalizing EBP. Worldviews Evid Based Nurs.

[42] Birken SA, Lee SD, Weiner BJ (2012). Uncovering middle managers’ role in healthcare innovation implementation. Implement Sci..

[43] Renz H, Autenrieth IB, Brandtzæg P, Cookson WO, Holgate S, von Mutius E, et al. Gene-environment interation in chronic disease: A European Science Foundation Forward Look. J Allergy Clin Immunol. 2011;128(6 Suppl):S27–49. 10.1016/j.jaci.2011.09.039 22118218

[44] Nonaka I, Konno N (1998). The Concept of “Ba” – Building a Foundation for Knowledge Creation. Calif Manage Rev.

[45] Metha V, Kushniruk A, Gauthier S, Richard Y, Deland E, Veilleux M, et al. Use of evidence in the process of practice change in a clinical team: a study-forming part of the Autocontrol Project. Int J Med Inform. 1998;51:169–80. 10.1016/s1386-5056(98)00113-0 9794332

[46] Thomas P, Hewitt J (2011). Managerial Organization and Professional Autonomy: A Discourse Based Conceptualization. Organ Stud.

[47] Gjersøe P, Morsø L, Jensen MS, Qvist P (2015). KOL-forløbsprogrammer har begrænset indvirkning på lægers tværsektorielle samarbejde. Ugeskr Laeger..

[48] Armstrong D, Ogden J (2006). The role of etiquette and experimentation in explaining how doctors change behaviour: a qualitative study. Health Illn.

[49] Morreim EH (2002). Professionalism and clinical autonomy in the practice of medicine. Mt Sinai J Med.

[50] Shaffer MA, Sandau KE, Diedrick L (2013). Evidence-based practice models for organizational change: overview and practical applications. J Adv Nurs.

[51] Nielsen B, Ward P (1997). Corporate management and clinical autonomy: the ethical dilemma in mental health. Aust Health Rev.

[52] Dogherty EJ, Harrison MB, Graham ID (2010). Facilitation as a Role and Process in Achieving Evidence-Based Practice in Nursing: A Focused Review of Concept and Meaning. Worldviews Evid Based Nurs.

[53] Kajermo KN, Nordström G, Krusebrant Å, Björvell H (1998). Barriers to and facilitators of research utilization, as perceived by a group of registered nurses in Sweden. J Adv Nurs..

[54] Hutchinson AM, Johnston L (2003). Bridging the divide: a survey of nurses’ opinions regarding barriers to, and facilitators of, research utilization in the practice setting. J Clin Nurs..

[55] Currey J, Considine J, Khaw D (2011). Clinical nurse research consultant: a clinical and academic role to advance practice and the discipline of nursing. J Adv Nurs.

[56] Adamsen L, Larsen K, Bjerregaard L, Madsen JK (2003). Danish research-active clinical nurses overcome barriers in research utilization. Scand J Caring Sci.

[57] Timmermans S, Kolker ES (2004). Evidence-Based Medicine and the Reconfiguration of Medical Knowledge. J Health Soc Behav.

[58] Weisz G, Cambrosio A, Keating P, Knaapen L, Schlich T, Tournay VJ (2007). The Emergence of Clinical Practice Guidelines. Milbank Q.

[59] Steffensen FH, Sørensen HT, Olesen F (1997). Impact of local evidence-based clinical guidelines – a Danish intervention study. Fam Pract.

[60] Cabana MD, Rand CS, Powe NR, Wu AW, Wilson MH, Abboud PAC, et al. Why Don’t Physicians Follow Clinical Practice Guidelines? A Framework for Improvement. JAMA. 1999;282(15):1458–65. 10.1001/jama.282.15.1458 10535437

[61] Mohan D, Rosengart MR, Farris C, Fischhoff B, Angus DC, Barnato AE (2012). Sources of non-compliance with clinical practice guidelines in trauma triage: a decision science study. Implement Sci..

[62] Bahtsevani C, Willman A, Stoltz P, Östman M (2010). Experiences of the implementation of clinical practice guidelines – interviews with nurse managers and nurses in hospital care. Scand J Caring Sci..

[63] Dreifuerst KT (2009). The Essentials of Debriefing in Simulation Learning: A Concept Analysis. Nurs Educ Perspect.

[64] Peters M, Ten Cate O (2014). Bedside teaching in medical education: a literature review. Perspect Med Educ.

