# Supplementary material for: Implementing research results in clinical practice- the experiences of healthcare professionals
Source: BMC Health Serv Res. 2016 Feb 10;16:48. doi: 10.1186/s12913-016-1292-y (PMC4748469; doi:10.1186/s12913-016-1292-y)
Supplement: Additional file 1: — Interview guide (translated from Danish). (PDF 70 kb) [file 12913_2016_1292_MOESM1_ESM.pdf]

Interview guide (translated from Danish)

**Themes and theoretical framework**	**Operationalization**	**Questions**
Opening questions		- What is your experience implementing research results in to everyday practice at the ward?<br>- Please describe how you have been involved in this.<br>- Have you ever been involved in implementation of research results? With a team? What was your role?
A package of implementation policies and practices established by an organization	Quality and quantity of necessary training in order to teach the staff to use the new evidence based initiative in practice.<br><br>Incentives to apply the initiative in practice – i.e. recognition and rewards.	- When you (or someone else) have decided to introduce new research results to your practice, what was your experience? What was done? Who did what?<br>- Is it your experience that someone is following the change in practice? And how is that done?<br>- Who is interested in whether things are actually being changed?
The climate for innovation implementation in the team or organization — i.e. the employees' shared perceptions of the importance of innovation implementation within the team or organization	Common understanding of the importance of the evidence based initiative in the team/organization.<br>Motivation – possibly through involvement.	- What is your opinion regarding changing your practice or the practice which you are part of when you come across new research results?<br>- Does it make sense to you when new results are to be implemented? How does it make sense?<br>- How do you experience the atmosphere when you are doing things in a different way than the usual based on new research results?
Managerial support for innovation implementation	The management's support for the implementation and the communication about evidence based initiatives is convincing, well informed	- Who typically informs you when an upcoming new initiative (based on research results) is to be implemented at the ward?<br>- What is your experience of how the management articulate new initiatives based on research results?<br>- Who typically articulates the vision/intention of the new practice?

		- If you are a manager yourself, how do you articulate the implementation of research results in your practice?
The availability of financial resources	Change of the requirements for personal resources.<br>Payment for training.<br>Time for learning in practice.<br>Adjustment of production demands while implementing the new initiative.	- Have you experienced an allocation of extra resources when the research based change of practice is taking place? (For example an increase in financial resources, competency development, extra hands).
A learning orientation: a set of interrelated practices and beliefs that support and enable employee and organizational skill development, learning, and growth	The employees' willingness to take risks and opportunity to experiment with the research based initiative.<br>The absence of a 'zero error culture'.<br>Change management with the emphasis on the vision of the initiative.<br>Own fallibility and the requirement of the employees' efforts in the implementation.<br>Acknowledgement of the employees' competences.	- When you implement new research results, how do you learn? Try to be very specific.
Managerial patience, i.e. long-term orientation	The management's patience and persistence in relation to the initiative.<br>Eye on long term outcomes as a supplement to the short term costs of the implementation.	- Have you experienced that research based initiatives have been left or maybe have faded out after a while? What happened?<br>- Do you experience that the management follows up on the new initiatives based on research results?<br>- If you are a manager, how do you follow the new initiatives based on research results?
